# DIGNiFI: Discovering causative genes for orphan diseases using protein-protein interaction networks

**DOI:** 10.1186/s12918-017-0402-8

**Published:** 2017-03-14

**Authors:** Xiaoxia Liu, Zhihao Yang, Hongfei Lin, Michael Simmons, Zhiyong Lu

**Affiliations:** 1 College of Computer Science and Technology, Dalian University of Technology, Dalian, Liaoning, 116024 China; 2 National Center for Biotechnology Information (NCBI), National Library of Medicine (NLM), National Institutes of Health, Bethesda, 20894 MD USA

**Keywords:** Orphan disease, Genetic disease, Protein-protein interaction networks, Eye disease

## Abstract

**Background:**

An orphan disease is any disease that affects a small percentage of the population. Orphan diseases are a great burden to patients and society, and most of them are genetic in origin. Unfortunately, our current understanding of the genes responsible for inherited orphan diseases is still quite limited. Developing effective computational algorithms to discover disease-causing genes would help unveil disease mechanisms and may enable better diagnosis and treatment.

**Results:**

We have developed a novel method, named as DIGNiFI (Disease causIng GeNe FInder), which uses Protein-Protein Interaction (PPI) network-based features to discover and rank candidate disease-causing genes. Specifically, our approach computes topologically similar genes by taking into account both local and global connected paths in PPI networks via Direct Neighbors and Local Random Walks, respectively. Furthermore, since genes with similar phenotypes tend to be functionally related, we have integrated PPI data with gene ontology (GO) annotations and protein complex data to further improve the performance of this approach. Results of 128 orphan diseases with 1184 known disease genes collected from the Orphanet show that our proposed methods outperform existing state-of-the-art methods for discovering candidate disease-causing genes. We also show that further performance improvement can be achieved when enriching the human-curated PPI network data with text-mined interactions from the biomedical literature. Finally, we demonstrate the utility of our approach by applying our method to identifying novel candidate genes for a set of four inherited retinal dystrophies. In this study, we found the top predictions for these retinal dystrophies consistent with literature reports and online databases of other retinal dystrophies.

**Conclusions:**

Our method successfully prioritizes orphan-disease-causative genes. This method has great potential to benefit the field of orphan disease research, where resources are scarce and greatly needed.

## Background

The US Rare Disease Act of 2002 defined a rare disease, also referred to as an orphan disease, as any disease that affects fewer than 200,000 inhabitants, equivalent to approximately 6.5 patients out of 10,000 inhabitants [1]. There are an estimated 8000 orphan diseases, and most of them are genetic in origin. Orphan diseases are a great burden to patients and society because they commonly afflict people early in life and persist throughout the lifetime. Some are even life-threatening [2, 3]. Discovering genes causing these diseases would unveil disease mechanisms and may enable better diagnosis and treatment. Unfortunately, our current understanding of the genes responsible for genetic orphan diseases is still quite limited [4]. In addition, even though the advent of next-generation sequencing has yielded great advances in our ability to collect data about patients with rare diseases, successfully sorting through this information to correctly identify the causal genes remains challenging [5]. Therefore, developing effective computational algorithms for the prioritization of candidate genes is a critical step in the research pipeline. Several earlier studies have shown that genes related to similar disease phenotypes tend to be functionally related, since genes execute their functions by interacting with one another through such means as sharing similar expression profiles, participating in signal tranduction mechanisms [6–8]. Moreover, researches have also shown that genes associated with phenotypically close disorders are likely to directly or indirectly interact with each other in the protein-protein interaction (PPI) network [8–11]. Based on this core concept and the principle of “guilt-by-association” [12], we propose a novel method to explore PPI networks and discover disease-causing genes.

Many computational approaches have been developed and applied to prioritize candidate disease genes from PPI networks [13–20]. Network-based disease-gene prioritization approaches can be broadly grouped into two categories: local similarity measures [14, 19] and global similarity measures [13, 15]. Local similarity measures consider whether two genes are directly connected or have a shorter path in the PPI network. However, many disease-causing genes don’t have such local connections and can only be connected through distant paths in PPI networks. For this reason, some groups have used global similarity measures obtained via various methods such as Random Walk with Restarts (RWR) to calculate the similarity between candidate genes and known disease genes [13]. Although these kinds of methods accurately capture global topological features of PPI networks, most of them require extensive computation. For these reasons, accurately identifying candidate genes through PPI networks remains challenging. In this paper, we propose a method, DIGNiFI, that calculates the topological similarity between two genes by considering local features based on shared Direct Neighbors and global features by Local Random Walks (LRW) [21].

Meanwhile, because human-curated PPI data contain various false-positive and false-negative interactions [22–24], integrating multiple resources such as gene expression profiles or GO annotations, is an alternative way to reduce the potential bias of using PPI data as a single resource for disease gene prioritization [18, 20, 25]. In addition, there is increasing evidence in genetic and molecular biology that protein complexes and pathways affect the interactions within groups of genes, perturbations of which lead to similar diseases [26]. In this work, we integrate GO annotations and protein complex data (DIGNiFI+SimBio) to further improve our proposed algorithm DIGNiFI. Also we apply these methods on an enriched network [27] that we generated by combining gene relations obtained based on proteins’ co-occurrences in biomedical literature and PPI interactions from biological experiments. All the algorithms were tested on 128 orphan diseases with at least five known genes downloaded from Orphanet [28]. The results demonstrate that our approach outperforms four state-of-the-art algorithms: VS [14], RWR [13], SPranker and SPGOranker [18]. VS method uses shortest paths to assess the closeness between two genes and RWR uses Random Walk with Restart to measure the distance between two genes, while SPranker uses shortest paths with weights to calculate the similarity between two genes and SPGOranker combines SPranker with GO functional annotations.

Furthermore, we apply our method to predict potential causative genes for several orphan eye diseases. Our top predictions include many genes with known associations with similar eye diseases and are consistent with literature reports and online databases. This case investigation of our method demonstrates its capability to discover causative genes for orphan diseases and suggests that other prioritized genes from our approach may be excellent candidates for further investigation.

## Methods

### DIGNiFI algorithm

The core assumption of disease gene prioritization from a PPI network is that genes that share topological similarities tend to be associated with phenotypically close disorders and may cause the same or similar diseases [8, 11, 29]. Such a “guilt by association” principle has been widely used to prioritize candidate disease genes. Hence, the most important task in using PPI network is measuring the similarity between known genes and candidate genes. In order to rank the candidate genes, we use two different ways to calculate the similarity: one is designed for directly connected genes and the other is for indirectly connected genes in the PPI network.

A PPI network can be presented as a graph *G*(*V,E,W*), where a set of nodes (*V*) denotes proteins together while a set of edges (*E*) denotes interactions between proteins with different edge weights (*W*). Given a protein *v*∈*V*, *Γ*_*v*_ represents the combination of *v*’ neighbors and *v*. From a topological view, if two genes share more common direct neighbors, those two genes are likely to be more similar. Hence, given a protein pair *v*_*i*_ and *v*_*j*_, we calculate the similarity between them by using Eq. 1.

1 $$  Sim\left(v_{i},v_{j}\right)= \left\{\begin{array}{ll} DN\left(v_{i},v_{j}\right)&\;e(v_{i},v_{j})\in{E}\\ LRW_{v_{i}v_{j}}(t)&\;otherwise \end{array}\right.  $$

The value of *DN*(*v*_*i*_,*v*_*j*_) is defined as:

2 $$ DN\left(v_{i},v_{j}\right)=\frac{\sum_{v_{k}\in\left(\Gamma_{v_{i}}\bigcap\Gamma_{v_{j}}\right)} w\left(v_{i},v_{k}\right)*w\left(v_{j},v_{k}\right)}{\sqrt{max\left\{ K_{v_{i}},K_{v_{j}}\right\}}}  $$

where *w*(*v*_*i*_,*v*_*j*_)=1 if *v*_*i*_ and *v*_*j*_ directly connect with each other or if *v*_*i*_=*v*_*j*_, otherwise *w*(*v*_*i*_,*v*_*j*_)=0. $K_{v_{i}}$ denotes the total edge weights that link to *v*_*i*_, and we use the maximum of *K* in order to depress the hub node effect. The value of $LRW_{v_{i}v_{j}}$ is derived by Eq. 3 according to [21].

3 $$  LRW_{v_{i}v_{j}}(t)=\frac{K_{v_{i}}}{M}{\pi}_{v_{i}v_{j}}(t)+\frac{K_{v_{j}}}{M}{\pi}_{v_{j}v_{i}}(t)  $$

where *M* is the number of links in the network and ${\pi }_{v_{i}v_{j}}(t)$ is the *v*_*j*_-th value of $\boldsymbol {\pi }_{v_{i}}(t)$ and $\boldsymbol {\pi }_{v_{i}}(t)$ is calculated by

4 $$  \boldsymbol{\pi}_{v_{i}}(t+1)=\boldsymbol{P^{T}\pi}_{v_{i}}(t)  $$

in which, ***π***_*x*_(0) is a *N*∗1 vector (N is the number of nodes in the network) which the *v*_*i*_-th is equal to 1 and others are 0. ***P*** is the transition probability matrix, with ${P}_{v_{i}v_{j}}=a_{v_{i}v_{j}}/k_{v_{i}}$ representing the probability that a random walker staying at node *v*_*i*_ will walk to *v*_*j*_ in the next step, where $a_{v_{i}v_{j}}$ equals 1 if *v*_*i*_ and *v*_*j*_ are connected, 0 otherwise. For two connected nodes, we use DN, which emphasizes the similarity of common direct neighbors, to calculate the similarity between them. In addition, we also consider that this approach may result in hub nodes receiving inappropriately high ranks, since they are connected to more nodes but are not necessarily the most directly similar genes. So we use maximum weight to penalize the hub nodes. At the same time, we use LRW to calculate the similarity between two indirectly connected nodes. One difficulty with general random-walk-based similarity measures is that they sensitively depend on parts of the network far away from the source nodes [30]. For example, the walker has a certain probability to go too far away from a source node to a target node even though they may in reality be close to each other. Using the LRW method can counteract this dependence and assign high similarity scores to the target node and the nodes nearby. Besides, the *t* step Local Random Walk algorithm has lower computational complexity than other random walk based algorithms and is suitable for scale and sparse networks [21]. As most disease genes connect with each other through calculable steps and as the PPI network is a large-scale yet sparse network [31, 32], LRW is a high-performance way to calculate the similarity between genes in the PPI network.

For a given disease *d*, if $S_{d_{k}}$ denotes the set of known genes, then the probability of a new candidate gene *v*_*c*_ to be a causal gene is evaluated by the sum similarity scores between all known genes and the candidate gene, as shown in Eq. 5:

5 $$  Score_{v_{c}}=\sum_{v_{i}\in{S_{d_{k}}}}Sim(v_{i},v_{c})  $$

**Fig. 1:**
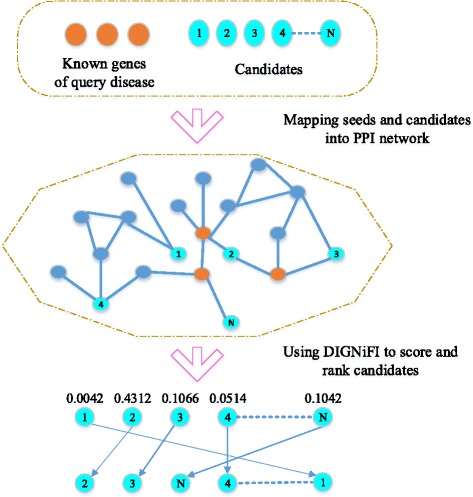
The flow chart of using DIGNiFI to prioritize diseases causing genes

After calculating the total score, we rank candidate genes of the given disease by their total scores. Figure 1 shows the flow chart of using DIGNiFI to prioritize disease causing genes for a query disease. As the similarity scores of *DN* and *LRW* can be pre-calculated, the complexity of ranking candidates genes depends only on the number of known genes when given a new disease.

### Integration with biological resources

It is well known that PPI data contain various false positive and false negative links. Therefore, integrating different data resources with PPI data should reduce the bias of using PPI data as a single resource and increase the ability of the PPI network to prioritize disease-causing genes. Recent research has demonstrated that genes with similar phenotypes often share common molecular signatures such as biological function, as measured by GO annotations [8]. Also, protein complex data is distinct from PPI network data, with clear, biologically relevant distinctions. For example, PEX26, PEX16 and PEX3 are three causal genes of Zellweger Syndrome. These genes don’t have any direct interaction in the PPI network, but do form a real protein complex. Hence, we integrate GO annotations and protein complex data to further improve our method.

#### Gene ontology annotation

The Gene Ontology project [33] provides a collection of well-defined biological terms for annotating genes and describing the characteristics of their gene products. GO annotation terms cover three separate fields: biological process, molecular function, and cellular components [34]. Many computational methods have used semantic similarity to calculate the similarity between two concepts in a taxonomy [35]. We employed a modification of a previous method [36] to calculate two genes’ semantic similarity by considering the number of common GO terms and how many genes the common GO terms have annotated. Specifically, we calculate two genes’ similarity based on their shared GO terms including biological process, molecular function and cellular component GO terms. For a given GO term, we define the annotation size of a GO term as the number of genes with that GO term. We then calculate the semantic similarity between two genes by the annotation size of their common GO terms. Thus, if two genes share a smaller annotation size of GO term, they are considered functionally more similar.

To describe the algorithm clearly, we first give some definitions. For a given gene *v*_*i*_, suppose it is annotated with *m* different GO terms. *S*_*k*_(*v*_*i*_) denotes a set of annotated genes with the GO term *g*_*k*_, whose annotation set includes *v*_*i*_, where 1≤*k*≤*m*. Suppose *n* is the number of common GO terms between gene *v*_*i*_ and *v*_*j*_, where *n*≤*m*. *S*_*k*_(*v*_*i*_,*v*_*j*_) denotes a set of annotated genes on GO term *g*_*k*_ whose annotation set includes both *v*_*i*_ and *v*_*j*_, where *k*≤*n*. Then, the semantic similarity of two genes based on GO annotations is calculated by the following formula:

6 $$ SimGO\left(v_{i},v_{j}\right)=-log\frac{min_{k}|S_{k}\left(v_{i},v_{j}\right)|}{|S_{max}|}  $$

where *min*_*k*_|*S*_*k*_(*v*_*i*_,*v*_*j*_)| is the minimum size of *S*_*k*_(*v*_*i*_,*v*_*j*_) and *S*_*max*_ is the maximum size of annotation among all GO terms.

#### Protein complex

Protein complexes are direct manifestations of the biologic interconnectivity of genes. It is likely that variants of genes whose protein products form complexes together may lead to similar disease phenotypes. Indeed, protein complexes have already been successfully used to predict disease-causing genes [37, 38]. However, these approaches overlook the information of the actual protein complexes by only using formed protein complexes based on topological properties (neighbors or densely connected subsets). Furthermore, these previous studies did not consider any of the unique characteristics of each protein complex. Many groups have demonstrated that dense subgraphs in a PPI network generally correspond to protein complexes [39, 40], and some studies show that if the nodes of a subgraph have more internal weight (or edges) than external weight (or edges), it will be more likely to form a group [41]. Thus the density and internal weight ratio of protein complex in a PPI network can be an index for the richness of protein interactions within the complex. In other words, proteins are more similar if they are in a more dense protein complex. Considering the two issues, we use the internal weight ratio [42] and the density to assign a network reliability score to an actual complex *C*_*k*_. The formula is shown by Eq. 7:

7 $$  Score(C_{k})=density(C_{k})*\frac{w^{in}(C_{k})}{w^{in}(C_{k})+w^{bound}(C_{k})}  $$

where, *w* ^*in*^(*C*_*k*_) is the total edges’ weight within a complex and *w* ^*bound*^(*C*_*k*_) is the total weight of edges that connect the complex with the rest of the network. The density of a protein complex *C*_*k*_ is defined as Eq. 8:

8 $$  density(C_{k})=\frac{2*|E_{C_{k}}|}{|V_{C_{k}}|*(|V_{C_{k}}|-1)}  $$

where $E_{C_{k}}$ and $V_{C_{k}}$ denote the edges and nodes in the complex respectively. Then, the *Score*(*C*_*k*_) can quantify the richness and reliability of the interactions with *C*_*k*_.

If two genes are in *M* same protein complexes, the similarity score between them is calculated as:

9 $$ SimCOM(v_{i},v_{j})=\sum_{k\in M}Score(C_{k})  $$

Finally, in order to integrate biological similarity (SimBio) with topological similarity (DIGNiFI), parameters *α* and *β* are used. The total score of a candidate gene with a known gene is calculated as:

10 $$ \begin{aligned} Sim\left(v_{i},v_{j}\right)=&\left(1-\alpha-\beta\right)DIGNiFI\left(v_{i},v_{j}\right)\\ &+\alpha SimGO\left(v_{i},v_{j}\right)+\beta SimCOM\left(v_{i},v_{j}\right) \end{aligned}  $$

Then, a candidate gene of a query disease is ranked by summing up the similarity scores between the candidate gene and the known genes of that disease.

## Results and discussion

### Data sources

Protein-protein interaction (PPI) data were downloaded from release 9 of the Human Protein Reference Database (HPRD) [43]. After removing duplicates and self-linked interactions, we obtained 9453 human genes and 36,867 interactions. Orphan diseases with causal genes were downloaded from Orphanet [28]. We selected all orphan diseases that had at least five causal genes found in the protein interaction network, resulting in 128 diseases and 1184 total genes.

The PPI network contains a number of false negative interactions because many interactions remain undetected by biological experiments. Nevertheless, the biomedical literature contains descriptions of many PPIs that are not catalogued in HPRD. For this reason in this paper, we also used the gene2pubmed dataset [44], which contains curated information about gene descriptions in PubMed articles, to test if combining protein relations mined from literature would be helpful for prioritization of disease-causing genes. We assumed that if mentions of different genes co-occur in an abstract, those genes are likely to have some types of interactions (direct or indirect). Note that we chose simple co-occurrence instead of advanced text mining techniques because relation extraction between bio-concepts remains challenging [45–47]. Thus to ensure high quality results from literature, we set a rather high threshold (the same pair needs to occur in 30 or more PubMed articles in this study), resulting in the selection of 16,118 gene interactions, 9600 of which overlapped with interactions in HPRD and 6518 did not. We merged these 6518 literature-mined interactions with the existing HPRD data.

### Experimental setting and evaluation criteria

Leave-one-out cross-validation was used to compare the proposed method with four state-of-the-art methods: RWR (with restart probability set to 0.8), VS, SPranker and SPGOranker (combining SPranker with GO functional annotations). Furthermore, we also compared DIGNiFI with LRW which calculates the similarity between two genes using Local Random Walk no matter whether these two genes are directly connected or not. In each round of cross-validation, one causal gene within an orphan disease, as the target gene, was removed. The remaining known causative genes for that disease were used as seed nodes, and each method was evaluated by the number of overall successes of ranking the target gene among top *k*. Specially, if the similarity score is equal to zero, then the rank of that candidate gene will be the size of the total candidate gene set. Considering the fact that the predicted top-ranked results are more important in practice, we utilized *k* values ranging from 1 to 10 in this paper, and the ratio of successful validation trails was used as the criterium for determining the “success rate”. In each cross validation trial, one target gene mixed with 99 randomly selected genes formed a set of 100 candidate genes. The step *t* of DIGNiFI is 3 as suggested by [48], and the default values of *α* and *β* in this paper are both to be 0.1.

### Experimental results

**Fig. 2:**
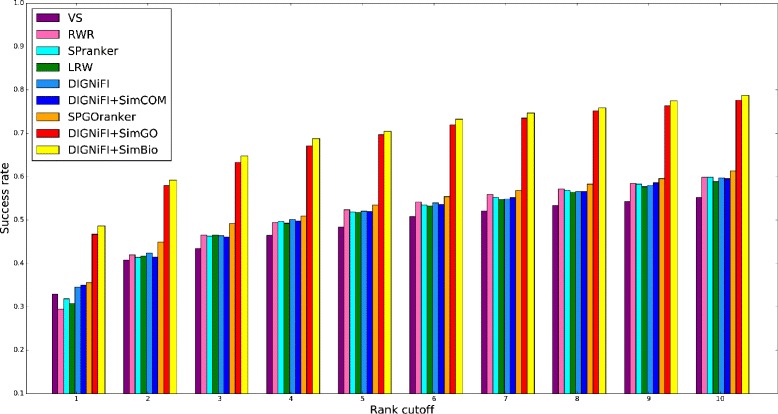
Performance comparison of different algorithms

The top-*k*-ranking results of each algorithm on the HPRD dataset are presented in Fig. 2. To perform these experiments, we manipulated the values of *α* and *β* to evaluate the contribution of each of these DIGNiFI modifications separately. Keeping *β* fixed as 0.0, only GO information is used (DIGNiFI+SimGO) and the best performance is obtained when *α*=0.1. While when *α*=0.0 only complex information is used (DIGNiFI+SimCOM) and the best performance is gained by setting *β* to 0.3. When *k*=1, the DIGNiFI+SimBio achieved the best performance with *α*=0.1 and *β*=0.1, its success rate is 48.65% (576/1184). Out of 1184 genes, DIGNiFI+SimGO successfully prioritized 553 (46.71%), and LRW+SimCOM successfully prioritized 414 (34.97%). By contrast, the success rates of DIGNiFI, LRW, VS, RRW and SPranker were 34.54% (409/1184), 30.74% (364/1184), 31.67% (375/1184), 29.48% (349/1184) and 31.84% (377/1184), respectively. Among all these five topological-feature-based methods, DIGNiFI performs best. The SPGOranker prioritization method, which also incorporates GO functional information, correctly predicted 421 genes, achieving a success rate of 35.56% and outperforming all others except DIGNiFI+SimGO and DIGNiFI+SimBio, which still surpassed SPGOranker by 34.36% and 36.81% respectively. Figure 2 shows that the DIGNiFI+SimBio consistently performs best throughout a range of *k* values from 1 to 10. Interestingly, these experiments also demonstrate that although VS performs better than RWR when *k* equals to 1, its performance only marginally improves with increasing *k* value. This is because VS only calculates two-node similarities by taking into account only proteins with direct interactions or shared neighboring nodes. If causal genes have more than one hop or step between them, VS is not able to identify their similarity. Furthermore, although ND-LRW+SimCOM does not correctly identify as many genes as DIGNiFI+SimGO and SPGOranker which both use GO information, it still outperforms the other methods that only use topological information, especially when *k*=1. These results demonstrate the effectiveness of the DIGNiFI algorithms and integration of biological resources improves the ability to detect disease-causing genes.

#### Analysis of the results when *k*=1

To further determine the performance of these methods, we analyzed the intersection of each result with *k*=1. The intersection results are shown in Table 1. Both the columns and rows represent the method used. The values in the table reflect the number of overlapping predictions generated by the corresponding column and row methods. DIGNiFI+SimBio understandably covers a high percentage of other methods since it identifies the greatest number of true disease-causing genes, including more than 100 different genes that were not detected by any other method. DIGNiFI+SimCOM with only 414 successful top-one predictions, surprisingly covers nearly 85% of the genes identified by other topological-feature-based methods. This indicates that both protein complex and GO annotations can help improve the performance for prioritization of disease-causing genes from another side. Interestingly, this table also demonstrates that different methods succeeded in identifying different genes. In order to determine if the results are significantly different from each other, we use two-tailed Student t test to compare the ranking results. The *p*-values between the ranking results from DIGNiFI+Simbio and other four methods are less than 0.05 (SPGOranker: 9.65*E*−26, SPranker: 1.51*E*−27, RWR: 3.58*E*−23, VS: 1.39*E*−70), which suggests that the differences between our methods and other state-of-the-art methods are not by chance.

**Table 1:** Results of each algorithms with *k*=1

Method	DIGNiFI	DIGNiFI	SPGOranker	DIGNiFI	DIGNiFI	SPranker	RWR	VS
	+SimBio	+SimGO		+SimCOM				
DIGNiFI+SimBio	576	479	331	332	329	324	276	306
DIGNiFI+SimGO	479	553	310	315	313	299	264	294
SPGOranker	331	310	421	328	331	315	280	314
DIGNiFI+SimCOM	332	315	328	414	349	301	296	334
DIGNiFI	329	313	331	349	409	302	288	331
SPranker	324	299	315	301	302	377	261	287
RWR	276	264	280	296	288	261	349	278
VS	306	294	314	334	331	287	278	375

#### Analysis of the effect of *α* and *β*

To analyze the effect of parameters *α* and *β*, we tested our prediction algorithms using values of *α* and *β* from 0.0 to 1.0 with 0.1 increments. The results with *k*=1 are shown in Table 2. When the parameters both equal to 0.0, the result is derived by DIGNiFI only. When *α*=0.0, the results are obtained by DIGNiFI+SimCOM while when *β*=0.0 the results are from DIGNiFI+SimGO. From the table, we can see that the results obtained from integrating PPI networks with biological information are much better than the results obtained from using topological similarity alone. Furthermore, the combined approach using both GO annotations and topological similarity outperforms the GO annotations approach. This indicates that functional and topological similarity contribute unique information to gene prioritization. Table 2 demonstrates that the success ratio for top one prediction is very low when using protein complex data alone. This may be due to the relatively small number of known protein complexes. When fusing protein complex data and topological similarity, although the results increase only slightly and some of them are not as good as using DIGNiFI, the best result of DIGNiFI+SimCOM show that it still can detect 82 different true disease causal genes than DIGNiFI+SimBio according to Table 1. These results show that even though protein complex data is not as rich as PPI network and GO annotation data, integrating real protein complex data still helps to improve prioritization of disease causal genes. This indicates that we may obtain improvements in the ability of prioritizing disease-causing genes with enhancement of protein complex data.

**Table 2:** Results with different values of parameters *α* and *β* with k=1

*α*	**β**										
	0.0	0.1	0.2	0.3	0.4	0.5	0.6	0.7	0.8	0.9	1.0
0.0	397	398	388	414	403	407	405	406	394	394	78
0.1	546	**576**	570	562	552	545	552	534	530	539	-
0.2	553	530	548	549	531	539	552	516	531	-	-
0.3	524	547	551	546	529	532	517	518	-	-	-
0.4	542	524	548	515	539	523	525	-	-	-	-
0.5	542	552	538	527	507	530	-	-	-	-	-
0.6	514	524	515	523	520	-	-	-	-	-	-
0.7	523	518	528	512	-	-	-	-	-	-	-
0.8	528	515	527	-	-	-	-	-	-	-	-
0.9	527	518	-	-	-	-	-	-	-	-	-
1.0	518	-	-	-	-	-	-	-	-	-	-

Bold is best result

#### The results on literature enriched PPI network

**Fig. 3:**
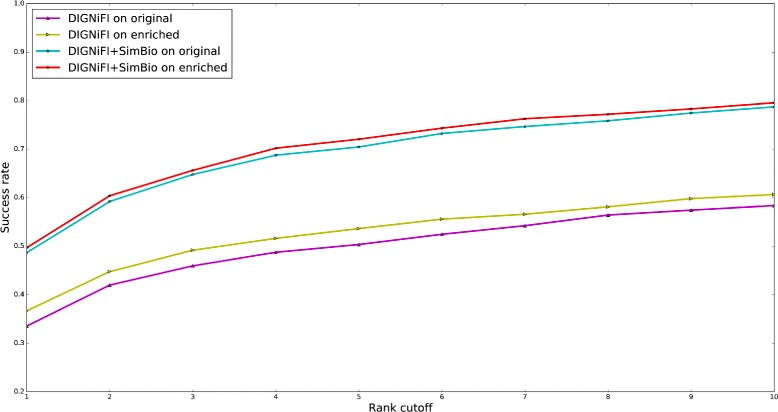
Performance comparison of different networks

For the reasons described previously in “Data sources” subsection, we used data from gene2pubmed to enrich the PPI network from HPRD. Figure 3 shows the best results of DIGNiFI and DIGNiFI+SimBio on both the original and enriched networks. The DIGNiFI and DIGNiFI+SimBio prediction algorithms perform consistently better on the enriched network than on the original network. These results indicate that the quality of PPI network affects the performance of gene prioritization. Since the performances of these methods improve after integrating even simple co-occurrence-based literature protein interactions into the PPI network, it is likely that further, systematic enhancements to the existing PPI network will result in continued improvements in disease gene prioritization performance.

#### Case study: prioritizing causal genes for inherited eye diseases

We further examined the capabilities of our method DIGNiFI+SimBio by predicting genes for a collection of four eye diseases, all of which involve degeneration of the retina: Retinitis pigmentosa (RP), Leber congenital amaurosis(LCA), Usher syndrome Type 1, and Congenital stationary night blindness (CSNB). All these diseases belong to a collection of rare, inheritable disorders called retinal dystophies. We include a brief description of each in Table 3. Orphanet contains lists of known genetic associations with these disorders, but another manually curated resource, the Retinal Information Network (RetNet), contains more comprehensive information about the genes related to these and other retinal dystrophies. We used the known genes listed in Orphanet as seed genes to predict additional genes that might be associated with these diseases. Specifically, for each disease, we ranked all genes in PPI network except the known genes in order to obtain the top ten predictions. We then compared our predictions with genes in RetNet to evaluate their validity.

**Table 3:** The top-ten predictions for four inherited retinal dystrophies

Disease/Syndrome	Description	# of Known genes	Top 10 predictions	Missing
Retinitis pigmentosa	A collection of blinding conditions involving bilateral degerneration of rod and cone photoreceptors and resulting in progressive vision loss. Symptoms generally begin in childhood with the loss of peripheral vision, and most patients are legally blind by the age of 40	44	BBS4, **GNAT1**, RORB, MYO7A, **MKKS**, **RBP4**, **PAX6**, RPGRIP1, CDH23, MYO5A	ARL3, HK1
Leber congenital amaurosis	A disease involving early degeneration of the retina as well as defects in the cornea and leading to severe vision loss in infancy	9	RBP4, **BBS4**, RGS9, **GNAT1**, **RPGR**, **USH2A**, CNGB1, RHO, RP1, ARL6	none
Usher syndrome type 1	A form of retinitis pigmentosa that involves hearing impairment in addition to vision loss	5	DFNB31*, USH2A*, CLIC5, **MYO3A**, **MYO15A**, KPTN, IQCB1, BBS4, RP1, NPHP4	CEP250, HARS
Congenital stationary night blindness	A non-progressive, inherited disorder of the retina that from birth causes a number of vision problems, including difficulty seeing in low light conditions	8	CNGA1, GUCY2F, CNGB1, RCVRN, RGS9, OPN4, RP1, **RPE65**, GNB1, OPN1LW	GNB3, RDH5

^*^ = overlap with RetNet gene; Underline = overlap with a different retinal dystrophy; Bold = literature support

As mentioned previously, a key assumption of PPI-network-based approaches to gene prioritization is that genes that share topological similarities tend to be associated with phenotypically close disorders. Therefore, we hypothesized that the genes that DIGNiFI+SimBio would predict from the Orphanet seed genes for a given retinal dystrophy would overlap with either the known genes listed in RetNet for that condition or the known genes for other retinal dystrophies. There were three possible outcomes of interest for each disorder we investigated. DIGNiFI+SimBio-predicted genes might (1) overlap with gene curations in RetNet for that disorder; (2) overlap with genes known to cause similar retinal disorders; or (3) not overlap at all with any known associations between genes and retinal dystrophes. It was also possible that DIGNiFI+SimBio might not prioritize genes that nevertheless had known associations in RetNet. Table 3 contains the results of this analysis.

The genes in the third column of Table 3 are hypothetical genes listed in order of their likelihood to be associated with a given disease as determined by DIGNiFI+SimBio. Two of the gene predictions generated by DIGNiFI+SimBio overlapped with known genes in RetNet. These genes, DFNB31 and USH2A, were the top two predictions for Usher Syndrome, Type 1. It is interesting to note that both genes are actually known to be associated with Usher Syndrome, Type 2. Although these specific hypotheses would thus not likely have practical utility for researchers or clinicians, they offer striking support for the validity of DIGNiFI+SimBio’s predictions. Furthermore, nearly all predicted genes for each retinal dystrophy overlapped with RetNet lists of genes for other retinal dystrophies. This overlap demonstrates that it is possible to use the topological features of PPI networks to identify functionally related and likely interdependent genes. The fourth column of Table 3 contains six genes that are listed in RetNet but not in Orphanet as having an association with the four retinal dystrophies we investigated. These genes were not in the top 10 predictions from DIGNiFI+SimBio but were in the PPI network. In investigating these genes, we found their ranks were 17(ARL3), 13 (HK1), 20(CEP250), 11(HARS), 33(GNB3) and 14(RDH5) respectively.

In addition to comparing our predictions with curations in RetNet, we also conducted a literature review for each disorder and its set of predicted genes. We performed this review by executing semantic searches for the relevant genes and diseases using entity-tagging tools in PubTator [49, 50]. Our search identified specific support for 10 of the 40 DIGNiFI + SimBio gene prioritizations, including at least one for each of the retinal dystrophies we studied. In some cases, the support was very strong. For example, PMID: 26900326 is a recent paper that reports an association between the gene MKKS, which has traditionally been associated with Bardet-Biedl syndrome, and retinitis pigmentosa. DIGNiFI + SimBio prioritized MKKS among the top ten novel genes from PPI most likely to contribute to retinitis pigmentosa. Although retinitis pigmentosa is one of the findings of Bardet-Biedl syndrome, the case reported in this study is unique because the retina findings resulting from this mutation caused retinitis pigmentosa in isolation of the other findings of Bardet-Biedle syndrome. Table 4 contains the results of the most relevant support that we identified in our literature investigation of the predicted genes.

**Table 4:** Literature support for genes predicted by DIGNiFI+SimBio

Disease	Gene	PMIDs	Comments
Retinitis pigmentosa	GNAT1	26472407	The first report of homozygous loss-of-function GNAT1 mutations leading to RP.
Retinitis pigmentosa	RBP4	23189188	Report of an association between the gene RBP4 and a form of early onset, progressive, autosomal recessive retinitis pigmentosa
Retinitis pigmentosa	MKKS	26900326	This gene is typically associated with Bardet-Biedl syndrome (BBS), but this report identifies a case of a MKKS mutation resulting in RP in the absence of any other typical features of BBS except polydactyly
Usher syndrome type 1	MYO3A	19390476	This gene is known to cause deafness, which is a distinguishing feature of Usher syndrome
Usher syndrome type 1	MYO15A	25404053	This gene is integrated in the ‘Usher interactome’, and although mutations of this gene have not been shown to lead to retinal dysfunction, they have been shown to cause hearing loss.
Leber congenital amaurosis	BBS4	22219648	Report of a novel variant of this gene causing LCA
Leber congenital amaurosis	RPGR	24981858, 20090203	RPGR is a receptor for RPGRIP1, and RPGRIP1 is known to associate with LCA
Leber congenital amaurosis	GNAT1	19672311	GNAT1 is a transducer molecule that leads to Bcl-2-mediated apoptosis of neurons in the presence of mutated RPE65
Leber congenital amaurosis	USH2A	18826961	This article discusses how USH2A is linked to LCA through the gene NINL (in the article, NINL is referred to as NLP).
Congenital stationary night blindness	RPE65	25307992	Review article that contains a table listing RPE65 as a known causative gene for CSNB

Although we identified support for many gene predictions through the literature and curations in RetNet, many genes remained without representation in any of these knowledge sources. These truly novel genetic hypotheses are strong leads for future research and discovery.

## Conclusion

In this paper, we propose a new algorithm, DIGNiFI, to prioritize causal genes for inherited orphan diseases. DIGNiFI considers both local and global features of genes in the PPI network and specifically uses Local Random Walks to identify global features. Leave-one-out cross-validation experiments with DIGNiFI show that DIGNiFI outperforms other algorithms that use topological features especially with ranking the top gene. We also explored the benefits of incorporating biological information from GO annotations and protein complex data into PPI network predictions. The resulting algorithm, DIGNiFI+SimBio, does indeed attain enhanced performance in predicting disease-causing genes. Furthermore, we reconstructed a PPI network by merging protein interactions from HPRD with protein interactions extracted from the literature using co-occurrence. Test results using DIGNiFI and DIGNiFI+SimBio on this text-mining enriched PPI network indicate not only that the sparseness of the PPI network limits gene prioritization but also that PPI relationships mined from biomedical literature can improve the quality of the PPI network and enhance gene-prioritization performance. Lastly, we use DIGNiFI+SimBio to predict genes involved in a set of four inherited retinal dystrophies. We found near-universal involvement of the predicted genes with retinal diseases and identified supporting literature for several of the hypothesized gene-disease associations. Taken together, these results demonstrate the relevance of our prediction method and indicate its potential utility in the field of orphan disease research, where resources are scarce and greatly needed.
